# *“This is streets ahead of what we used to do”:* staff perceptions of virtual clinical pharmacy services in rural and remote Australian hospitals

**DOI:** 10.1186/s12913-021-07328-w

**Published:** 2021-12-04

**Authors:** Julaine Allan, Emma Webster, Brett Chambers, Shannon Nott

**Affiliations:** 1 School of Health and Society, University of Wollongong, Wollongong, NSW 2522 Australia; 2 School of Rural Health, University of Sydney, Dubbo, Australia; 3 Western NSW Local Health District, Dubbo, Australia

**Keywords:** Clinical pharmacy, Healthcare access, Internet pharmacy, Pharmacist, Rural and remote, Staff perceptions, Telehealth, Telepharmacy, Virtual pharmacy

## Abstract

**Background:**

The use of medications is the most common intervention in healthcare. However, unsafe medication practices and medication errors are a leading cause of injury and avoidable harm in healthcare systems across the world. A Virtual Clinical Pharmacy Service (VCPS) was introduced in rural and remote New South Wales public hospitals to support safe and effective use of medications. In this model clinical pharmacy services are delivered via a telehealth cart at the patient’s bedside and through electronic medical and pharmaceutical record systems. The aim of this research was to understand healthcare staff perspectives of the VCPS and identify areas for improvement.

**Methods:**

A qualitative approach informed by Appreciative Inquiry was used to investigate healthcare staff perceptions of the VCPS. Focus group discussions (*n* = 15) with hospital staff and medical officers were conducted via videoconference at each study site. Focus groups explored issues, benefits and barriers 3 months after service implementation. Transcribed data were analysed using thematic analysis and team discussion to synthesise themes.

**Results:**

Focus group participants identified the value of the VCPS to patients, to the health service and to themselves. They also identified enhancements to increase value for each of these groups. Perceived benefits to patients included access to specialist medication advice and improved medication knowledge. Staff valued access to an additional, trusted workforce who provided back-up and guidance. Staff also reported confidence in improved patient safety and identification of medication errors. Enhanced compliance with antimicrobial stewardship and hospital accreditation standards were beneficial to the health service. Suggested improvements included extending virtual service hours and widening patient eligibility to include aged care patients.

**Conclusions:**

The VCPS brought a positive, collegiate culture regarding medications. Healthcare staff perceived the VCPS was effective and an efficient way for the health service to supply pharmacy services to smaller hospitals. The ease of use, model of delivery, availability, local knowledge and responsiveness of highly skilled pharmacists was the key to user satisfaction.

**Trial registration:**

ANZCTR ACTRN12619001757101, 11/12/2019.

**Supplementary Information:**

The online version contains supplementary material available at 10.1186/s12913-021-07328-w.

## Background

Access to safe and effective medications is a key target of the UN sustainable development goals [1] as the use of medications is the most common intervention in healthcare [2]. Unsafe medication practices and medication errors are a leading cause of injury and avoidable harm in healthcare systems across the world [3]. To achieve the international goal of reducing severe avoidable medication related harm by 50% in 5 years, all countries need to ensure medications are correctly supplied and used [3]. In Australia, where medication errors are the fourth most commonly reported incident in hospitals, medicine safety has been declared a National Health Priority Area [4].

Clinical pharmacists are utilised to improve medication safety in hospitals [5]. They fulfil key clinical functions including taking a medication history, medication reconciliation on transitions of care, medication review, provision of up-to-date medication lists and counselling to patients. Metropolitan areas are well served by clinical pharmacists, however in smaller hospitals particularly in rural and remote areas, the medication management tasks are allocated to medical or nursing staff [5, 6]. The absence of pharmacists with specialist medication knowledge makes smaller hospitals more vulnerable to medication errors [7]. It also becomes challenging (if not impossible) for smaller hospitals to meet national accreditation standards for medication safety without pharmacist availability [8].

Recognising the above challenges and traditional employment patterns, virtual health care has the potential to increase access to pharmacy services in rural and remote hospitals. Virtual care includes the use of telephone, videoconference and remote monitoring to connect patients with healthcare professionals [9]. As technology has improved and become more accessible, opportunities for virtual care have increased and different delivery strategies tested [10]. Ease of use, reliable equipment, collaboration and flexible and responsive working practices support virtual care delivery [11–13]. Whereas concerns about staff and patient relationships, low expectations of outcomes, impact on staff autonomy or credibility, and problems with technology have been established as barriers to use of virtual care regardless of its availability or patient need [10, 12, 14].

Virtual clinical pharmacy services are an evolving model of care [15]. Most studies focus on outpatient clinics and chronic disease management such as cardiology, diabetes and hypertension [16–18]. Research on virtual pharmacy models thus far has focussed on pilot and feasibility studies describing service implementations [19] or evaluation of outpatient clinics demonstrating improvements in surrogate end points [20, 21] Few studies have evaluated the role of hospital staff in the implementation of virtual pharmacy services.

Healthcare staff are critical to implementing and embedding new clinical services [22]. This is also true for virtual models of care, where, staff are vulnerable to increasing work demands and new or adapted tasks that are rarely considered in implementation planning [23]. Even when a healthcare gap is filled, change fatigue can leave health care workers, in particular nurses, feeling burnt out and apathetic [24], resulting in increased safety risks and increased medication errors [25].

Given that successful virtual health service implementation relies on staff support and participation, this study aims to understand hospital staff perspectives of the implementation of a Virtual Clinical Pharmacy Service (VCPS) in rural and remote New South Wales (NSW).

## Methods

A qualitative approach informed by Appreciative Inquiry [26], was used to investigate healthcare staff perceptions of the VCPS and guide service learning. Appreciative Inquiry has its roots in organisational development and provides a theoretical lens to focus on the strengths of the VCPS and visioning improvements by concentrating on discovery (valuing current strengths), dream (envisioning what might be), design (discussing enhancements) and destiny (applying innovations) [26]. Focus groups were chosen for data collection because they are a proven forum to examine shared health service staff experiences of workplace processes and events [27]. Focus group questions explored benefits, issues, barriers and overall acceptability of the service (Additional File 1).

### Study context and intervention

This study was situated in rural and remote NSW, Australia. Hospital care in the area is provided through a network of rural referral hospitals, small hospitals, multipurpose facilities and nurse-only remote clinics. The estimated population of 278,759 people is geographically dispersed over 433,379 km^2^ [28]. Residents experience higher than average annual mortality rates and potentially avoidable death rates compared to the rest of NSW [28]. A VCPS was established in eight small hospitals that did not have onsite pharmacists [29]. The VCPS was implemented between April and November 2020 and was available to emergency and acute inpatients. Hospitals included in the study had between 12 to 24 inpatient and emergency beds and recorded an average of twenty patient discharges per month. The goal of VCPS was to provide comprehensive clinical pharmacy services virtually to increase the safe and effective use of medicines and improve compliance with national accreditation standards. The service focussed on the core roles of a hospital pharmacist including medication reconciliation, medication review and patient education delivered virtually by utilising existing videoconferencing technology, the electronic medical record and electronic medication platforms. Patients were referred into the service by staff or proactively reviewed by a virtual pharmacist on admission.

The VCPS was resourced with a dedicated project manager and clinical lead who conducted local engagement prior to implementation. A structured implementation process was employed at each site which included an in-person visit, staff education on the role of the service, process and technology. One site was implemented monthly as part of the stepped-wedge study design which allowed learnings to be incorporated into the implementation plan. Engagement continued post start-up through multidisciplinary team meetings, monthly service level meetings, education sessions and newsletters. Pharmacists who delivered VCPS services all had experience working in the health district.

### Data collection

Focus groups were held 3 months after the implementation of VCPS to provide adequate time for staff to attain a working knowledge of the service. Consistent with Appreciative Inquiry, questions focussed on employee experience of the VCPS implementation, appreciation of benefits to patients and staff and design for future service improvement. Staff employed by the health service who regularly interacted with VCPS were invited to participate in a focus group at their worksite at least a week prior to the session. Two groups were held at each site, one for medical staff and one for nursing staff. The nursing focus group also included site managers who were nurses. Two further groups were held with Allied Health staff who worked across several sites; and with VCPS Pharmacists. The date for each site was negotiated with the site manager. Groups were held mid-afternoon at shift changeover time to allow morning and afternoon staff to attend or at night. A staff email address list was obtained by the project manager (BC) who emailed the focus group details and participant information sheets.

Focus groups at each site were organised by the project manager (BC) and conducted by one researcher (JA) who was independent from the service to prevent potential response bias. Focus groups were held over videoconference using *Pexip* software, an online encrypted teleconferencing and videoconferencing system used on all sites since 2016 for meetings and patient care.

Each virtual focus group commenced with an explanation of the research followed by participants reiterating their consent on audio recording. Any person not wanting to participate or be recorded was asked to leave. The focus groups were audio recorded and transcribed verbatim by an independent transcription company.

### Data analysis

**Fig. 1:**
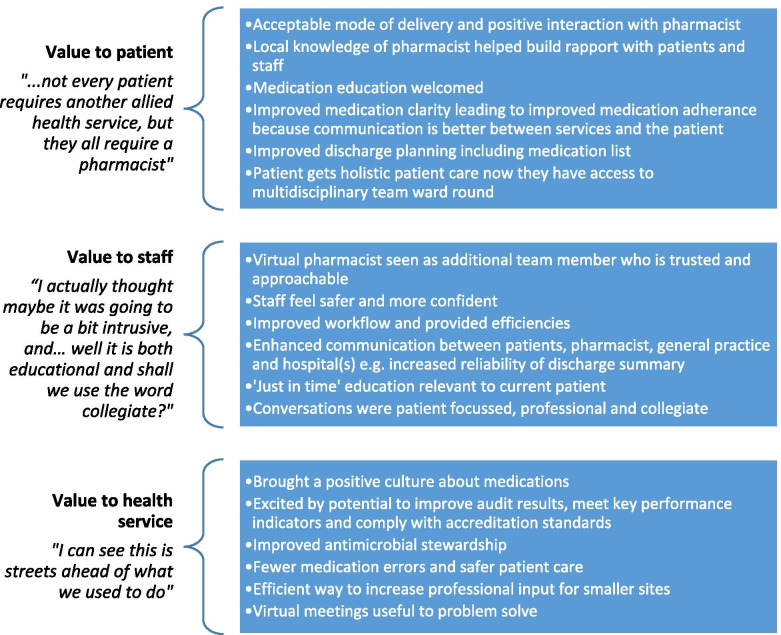
What participants appreciated about the Virtual Clinical Pharmacy Service

**Fig. 2:**
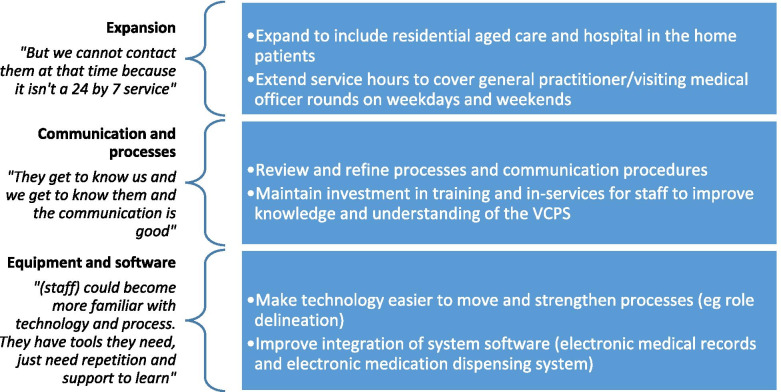
Envisioned enhancements to the Virtual Clinical Pharmacy Service

Transcripts were analysed by following established thematic analysis steps [30]. Two researchers experienced in qualitative methods (JA, EW) independently open coded data from the first five sites. The researchers compared and discussed initial codes and themes. Coding criteria were reviewed and refined as additional data was added. Themes were arranged and rearranged, with final themes informed by Appreciative Inquiry and agreed by consensus between three researchers (JA, EW, BC). Appreciation of the value of the VCPS to the patient, the staff and the health service reflect the strengths of the service (see Fig. 1) on which further enhancements can be built (Fig. 2).

## Results

Fifteen focus groups were conducted between July 2020 and April 2021 (Table 1). In total 67 staff participated in the focus groups including nursing (64%), doctors (12%), managers (12%), allied health (6%) and pharmacy (6%). While no one declined consent, some staff were unable to complete the entire focus group session due to competing clinical duties (*n* = 5). One focus group was conducted over the phone owing to telehealth equipment being used for clinical work.

**Table 1:** Staff who participated in Virtual Clinical Pharmacy Service focus groups

Focus Group Number	Hospital Location	Nurse n (%)	Manager n (%)	Doctor n (%)	Pharmacist n (%)	Allied Health n (%)	Entered late or left early n (%)	Number of participants n (%)
1	Site 1	6	2				1	8
2 & 3	Site 2	6		1				7
4 & 5	Site 3	9		1			1	10
6 & 7	Site 4	4	1	2			2	7
8 & 9	Site 5		2	1				3
10	Site 6	8						8
11 & 12	Site 7	5		3			1	8
13	Site 8	4	2					6
14	VCPS providers				4			4
15	Allied Health/ Other	1	1			4		6
Total		43 (64)	8 (12)	8 (12)	4 (6)	4 (6)	5 (7)	67

Themes reflect the influence of Appreciative Inquiry. The first section describes staff perceptions of the value of the Virtual Clinical Pharmacy Service to patients, to staff and to the health service. These themes encapsulate the current strengths of the virtual service (summarised in Fig. 1). The second section is focussed on envisioned enhancements to build on the strengths and maximise potential of the VCPS (summarised in Fig. 2). Quotes are presented from focus groups (FG) including the group number which can be cross-referenced to Table 1.

### Value to patient *“...not every patient requires another allied health service, but they all require a pharmacist” FG8*

Staff perceived patients were very accepting of the virtual pharmacy service and were comfortable using it*.* Staff described most patients as being able to engage with the virtual pharmacist in a similar way to in-person engagement.

“I wondered how some of the older generation would go but they've spoke nothing but high praise of it… I think because [virtual pharmacist] does such a brilliant job of interacting with them and talking to them about their medications, they've just embraced it.” FG 8

The technology was reliable, and most patients could see and hear the consultation.

*“The patients are really receptive to it. The whole model. They really – because the screens are so big on the Wallie* [telehealth cart] *usually, and it’s really good. They can turn it up loud and go for it.” FG15*

The interpersonal skills of the virtual pharmacist were critical in establishing rapport with the patient. Patients and carers were receptive to medication education they received and felt comfortable asking questions of the virtual pharmacist.

*“I was watching a consult just earlier today, and* [virtual pharmacist] *said, okay, I'm going to quiz you- to the partner - because she's in charge of the medication. So she did - it's just a very nice and fun, free kind of thing.” FG4*

Staff reported how valuable it was for virtual pharmacy staff to have local knowledge as this helped build rapport with patients and grasp nuances which were important for discharge planning such as the size of the town the patient was from and the distance from a major centre.

*“Actually, that’s been one of the good things is that some of the pharmacists involved in our project were based in Dubbo. So, they knew our hospital and probably some of the others [*hospitals*] really well. So, if the patients said, oh, I’m from, say, Eumungerie, if you’ve got a doctor from the middle of Canada saying, I have no idea where that is, but the pharmacist did know where that was. So that’s actually been a positive thing.” FG13*

Staff noted the more informed the patient (and their carer) was about their medications, the more likely they were to take the medications. Staff described the proactive role virtual pharmacists took in discharge planning such as organising or updating dose administration aids and ensuring the patient received a patient friendly medication list prior to discharge. Patient safety was seen to be enhanced by these improvements in communication between the hospital staff, patient, community pharmacy and general practice.

*“...it was all just word of mouth; the doctor would tell the patients and us at the handover what the [medication] changes were* [on discharge] *and then he'd do the scripts and then they'd leave with the scripts even if we don't do it* [medication list] *- but at least this way, if there's something lost in that, if they've actually got a physical piece of paper that says, this is the medications you're meant to be on, it's much safer.” FG13*

Staff identified the positive value to the patient by pharmacist involvement in the multidisciplinary team ward round. Staff felt all patients benefitted from the pharmacist involvement and identified that this was the first time they had been able to offer holistic patient care.

*“… the MDT* [multidisciplinary team ward round] *is just the icing on a beautiful cake because for the only time in my 24 years of working at* [remote*] Hospital we actually do have a proper multidisciplinary round with the patients in the centre… So, for once I can actually say that holistic approach to patient care is 100 per cent...” FG8*

### Value to staff *“I can’t say how well we’ve embraced it and how much we love having this service. Not only just for us but for the patients, for the doctors, it’s been great.” FG8*

Staff viewed the virtual pharmacist as an additional team member who was trusted and approachable. They valued the double checking, reminders, back-up and guidance provided as they felt it led to safer practice, fewer medication errors and improvements in patient safety. Their experience of interacting with the service was efficient, but they also described efficiencies it brought to their own work.

“Easier, accessible, because they do medication reconciliations, they can see everyone, so there's efficiencies there, scanning what the orders are and any drug interactions. So it's quite, well, I think, useful and efficient and there's a patient safety focus as well” FG13

The VCPS provided regular formal education sessions to each site. However, focus group participants described numerous occasions where ‘just in time’ medication education relevant to current patients was extremely valuable. For example;

*“I wanted to know why and what could we do better and what could we do differently, like was this appropriate for this patient, and they were really, really helpful. One* [virtual pharmacist*] was able to have a quick chat with me and explain it and I felt I had a much better understanding of what was going on and why the patient - why we were doing what we were doing.” FG10*

Learning more about medications made clinicians more interested in how medications might be relevant to the care of a patient.

“… just getting that bit of education as we’re going, going “What does that drug do?” or “What’s that indication?”.... Because I mean pharmacy sort of stuff… – the names of different medications, all the different things. I can’t keep up with all those changes... It’s good.” FG15

Participating in ward rounds or patient medication reviews was also an education opportunity for nursing staff;

“Listening to them give the patients education. We’re learning at the same time as everyone else”. FG15

In spite of some having initial misgivings, focus group participants unanimously reported benefits from pharmacist involvement.

“I actually thought maybe it was going to be a bit intrusive, and… it didn’t take very long to see that it was a great help… well it is both educational and shall we use the word collegiate?" FG 9

Some nursing staff reported pharmacist communications also had a positive effect on their communication with medical staff as it had removed the perception nurses were challenging a doctor’s authority.

*“…but the conversation between* [virtual pharmacist] *and clinician is very much a professional, informative conversation. But it always brings it back to the patients. Whereas I'm going to say before with not having that input from pharmacy it was very much seen as a challenge between doctor and nurse.” FG8*

Virtual pharmacists reported less distractions from providing clinical services, time saved by not needing to change work locations within hospitals, improved continuity of care for patients and overall high job satisfaction.

“…I think that like as a job this is much more rewarding and much more – like I feel more satisfied doing this job. …I probably have had a like reinvigoration of what we can do and how we can benefit our patients. So I think it's a rewarding job… FG14

### Value to health service “*I can see this is streets ahead of what we used to do” FG13*

Staff reported the VCPS brought a positive culture about medications and were excited about the potential to improve hospital audit results, enhance compliance with key performance indicators and meet accreditation gaps.

“From a management side, when I'm doing my audits it's very, very helpful to have the pharmacist that's been in there and done the initial medication, best medication history…and yeah, the reconciliation then at the end. It has improved our audit results a lot…” FG1

A key aim of the VCPS to improve antibiotic prescribing was also noted.

“The other thing that has really improved is the antibiotic prescribing… it's far more in line with the therapeutic guidelines.” FG1

Staff were convinced of the potential of the virtual service to improve patient safety. For example a doctor stated; *“I think it’s brilliant for its safety. It does improve safety. It has to. I mean how can it not?”(FG7).* However, some participants identified medication errors had increased because more errors were being identified;

“I wouldn’t necessarily say the medication error rate is going up, just that the detection of the errors is going up.” FG9

Because of the VCPS, responsibility for identifying medication errors was shared between nursing and pharmacy staff;

*We’re having a higher incident of reporting… I don’t see it as a negative aspect. The pharmacists also are picking up on errors and IIMSing them* [reporting] *and putting them through too. So that’s been a good thing and creates a safer culture here.” FG4*

Investment by the health service in effective technology was critical to the success of the VCPS. Not only was there ease of use and accessibility for patients but the electronic medical record and the electronic medication management (eMeds) system facilitated timely communication between doctors, nurses, pharmacists and patients. For example;

*“The virtual pharmacist suggestions, comments, etcetera, are there* [in electronic medical record], *and it’s pretty hard to miss. I mean – so, the degree of interaction is much more than I’ve* [doctor] *had previously with hospital-based pharmacists. FG9*

### Envisioned enhancements to the VCPS

Focus group participants were asked to suggest improvements to the VCPS that would make it easier to use for patients, staff and the health service. Suggestions included having the VCPS available to more patients, for longer hours including weekends, and continuing to work on communication including maintaining investment in training and in-services for staff to improve knowledge and understanding of the VCPS (Fig. 2).

### Expansion

Focus group participants were asked about how the virtual pharmacy service could be improved. Access to the VCPS was limited to hospital inpatients Monday to Friday 8 am to 4.30 pm during the trial. Staff felt the service would also be beneficial for other patients attached to the facility such as residential aged care patients or those getting hospital services at home.

*“I think if they were going to look for improvements, they would be looking at all patients, whether they're TACP* [Transitional Aged Care Package] *or subacute or acute for that matter”. FG3*

Extending the hours of operation would also make the VCPS more accessible for Visiting Medical Officers who also work in their own general practices. General practitioners tended to conduct hospital rounds early in the morning and late in the evening as well as on weekends. This resulted in less synchronous communications with the pharmacist instead relying on the eMeds system and medical records to share information and recommendations without the opportunity to clarify via phone or video call.

*“We’re contractors* [Visiting Medical Officers]. *We’re not employees. That’s the other thing. I felt we were treated like employees not contractors. We run - in solo rural practice I’ve got commitments - a lot of external commitments independent of the hospital” FG7*

### Communication and processes

Participants placed high value on relationships and trust they had with other service providers and with patients. Some local doctors had ongoing relationships with many patients outside the hospital and perceived their knowledge of patient’s history including community pharmacy support was not taken into account with the VCPS;

*“There are community patients who are going to go back into the community and then be community patients again which I will then manage myself again. There was a lot of double handling in that* [instance]*.” FG7*

Nursing staff also noted that communication between doctors and pharmacists could be improved to ensure optimal patient care;

*“I just find that me personally I have just run today from either the* [virtual] *pharmacist to the doctor, to the phone and back again.”* FG11

Implementing new procedures invariably changes tasks and potentially impacts staff roles in different ways. Some staff described challenges during the start-up phase of the VCPS when they were not sure how to use the equipment including when and why to call the pharmacist. Because many staff in small hospitals work part time they had missed the initial information and education sessions about the VCPS or did not always get an opportunity to interact with the pharmacists if they worked weekends and nights most of the time;

“Well, I didn't do the education, so I don't know if it was covered but up until now it had never occurred to me that I could use a virtual pharmacist to get answers to questions about medication.” FG13

While processes were mostly perceived as user friendly and effective some focus groups suggested refining procedures and systems;

*“But that is the helpline number which takes a lot of time. A direct number it will be a bit quicker.”*FG3

Some participants suggested improving the timeliness of medication lists on discharge because of how quickly patients wanted to leave the hospital;

*“It's nothing actually to do with the actual pharmacy per se it's just they* [patients] *just want to go home, and they're not being held up anymore. Take the cannula out, I'm going home” FG6*

### Equipment

Some staff perceived the task of taking the equipment to the patient’s bedside to be an administrative responsibility not a nursing one and described resenting the ‘interference” with their usual duties.

“So, it very much relies on the nursing staff on the ground to have to, you know, take the VC to the patient and talk to the doctor” FG 6*.*

However, without exception all focus groups reported these implementation challenges had been resolved or worked around although one group suggested making the Wallie [telehealth cart] easier to move would improve their experience of the VCPS.

### System software

One group reported some ongoing difficulties with the electronic medical record and the eMeds system. Improvements in the software so that it was consistent across different wards (intensive care unit to emergency department/general wards) would make for a safer system. The work of the virtual pharmacist picked up errors between these systems but staff felt improvements in the systems would deliver benefits to the health service in terms of more efficient use of time and improvements to patient care.

*“When their meds were charted the [admitting] doctor looked at the medication list but that was actually one from April. This was to do with a patient being in ICU* [Intensive Care Unit] *and when they're in ICU they're on a different software system. Like in the general ward they're on power chart and so that's how we find their medication through power chart. But when they're in ICU it's called something else. So, we actually don't have access to it. We can't even see it”FG6*

## Discussion

This study provides insight into staff perspectives of a Virtual Clinical Pharmacy Service implementation. Focus groups with healthcare staff across eight rural and remote hospitals in western NSW found the VCPS brought a positive, collegiate culture regarding medications. The service was acceptable, efficient and effective with few barriers to implementation. Staff explained how supporting processes, technologies and human factors made the VCPS feasible; and identified areas for further refinement. These findings demonstrate the virtual delivery of clinical pharmacy services to small rural and remote hospitals was successful in a real-world setting.

Staff confidence and trust has proven critical in the successful operationalisation of virtual care [14, 18]. Staff in this study described a positive culture around medications, safer practice, medication errors and improvements in patient safety. Focus group participants reported flow on effects in improving hospital audits, antimicrobial stewardship, key performance indicators and compliance with accreditation standards. The introduction of the service was also seen to provide more holistic patient care through multidisciplinary care rounds and improved communication with patients, staff and the hospital in general. Staff provided suggestions to improve the service through refining some processes to improve integration with other teams, communication with doctors, expanding opening hours and increasing access to more patients.

The high acceptability of this service may reflect the strong focus on implementation and change management including site visits and education sessions. Many studies cite ease of use, reliability and support of telehealth equipment as key facilitators for virtual care uptake and delivery [10, 11]. These factors are key components of the VCPS, with staff reporting reliable technology, a well-organised service and efficient and easy to use processes. Some staff described challenges using equipment and understanding the process during the start-up phase however this resolved with practice. The success of the VCPS is likely explained in-part by the long history of telehealth programs at most study sites resulting in staff familiarity with technology and virtual models of care. High reliability has been achieved through a significant investment in digital infrastructure and technology and a dedicated telehealth support team. These would be important considerations for other providers looking to implement virtual healthcare.

Positive working relationships between health professionals combined with systems that support communication are essential factors in healthcare delivery [2, 7, 13]. Communication, collaboration and professional relationships were recurrent themes throughout the focus groups and also proposed as a potential area for improvement, particularly between virtual pharmacists and medical officers. VCPS pharmacists were selected based on their communication skills, an area recognised early by the service as essential for success. Staff also highlighted the importance of the electronic medical record and eMeds system as important enabling factors in communication across sites and between pharmacists and doctors.

Local staff at hospitals are critical for the successful uptake of a virtual pharmacy program [12, 25]. Staff engagement facilitated the VCPS in spite of some start-up problems. Small local teams, part time employees, high staff turnover and local doctor schedules created challenges in implementation that was resolved over time as the site became familiar with using the service. Initially there were concerns over additional workload with setting up telehealth consultations but there were no reports of on-going task shifting once processes were established [23]. Staff identified potential time saving with the pharmacist performing medication reconciliation and medication lists which would otherwise be performed by nurses. Change fatigue was not identified as a factor in VCPS implementation although some tasks, such as taking telehealth equipment to the bedside, were considered more appropriate for administration staff [22, 24].

This is the first multicentre study to examine healthcare staff perceptions of an inpatient virtual pharmacy service in detail. The findings are significant as they demonstrate inpatient virtual pharmacy services are acceptable to staff and offers a mechanism to provide access to clinical pharmacy in rural and remote hospitals. This research has potentially wide reaching impact for the provision of pharmacy services to rural and remote Australian hospitals as well as hospitals worldwide with limited or no access to clinical pharmacy services. It also provides an alternative to other proposed models of rural clinical pharmacy delivery such as hub and spoke outreach models and external contracted providers [6]. High job satisfaction reported by pharmacists may also help with retention of qualified staff in rural areas.

Despite consistent findings there are several limitations to consider. Focus groups were short and scheduled at change of shifts when staff availability was at its highest. However, some staff may have limited their contribution because they wanted to go home or start their shift. As only a sample of staff at each site participated it is possible that some staff may have avoided the focus groups. Staff members who were new, work night shifts, weekends, part-time or casual may have had limited opportunities to work with VCPS over the 3 months prior to the focus groups. Further, on-line focus groups have been found to limit the researcher’s ability to establish rapport and facilitate open discussion potentially limiting responses in this study [31]. However, support for the VCPS and the way it was run was consistent across all focus groups.

The VCPS has filled a critical gap in pharmaceutical care in participating rural and remote hospitals. The use of Appreciative Inquiry as a theoretical approach to the research gave a positive platform to highlight the value of the new service to the patients, staff and the health service and welcomed staff opinions to further enhance the service. The successful implementation has resulted in widespread organisational support and expansion of the service to all hospitals in the local health district without an onsite pharmacy service.

## Conclusions

The VCPS are an acceptable model of care to improve medication management in small rural hospitals. The service was perceived as an effective clinical service that works alongside onsite and other virtual clinicians to provide comprehensive clinical care. The VCPS brought a positive, collegiate culture regarding medication safety and effective use of medicines. The ease of use, model of delivery, availability and responsiveness of highly skilled pharmacists was the key to user satisfaction. We have demonstrated the translation possibilities of virtually delivered clinical pharmacy services across rural and remote health facilities in Australia and potentially worldwide.

## Supplementary Information

**Additional file 1.**

## Data Availability

The datasets generated and analysed during the current study are not publicly available due to participants and sites being easily identified in transcripts but are available from the corresponding author on reasonable request.

## Abbreviations

ANZCTR: Australian and New Zealand clinical trials registry
eMeds: Electronic medication management system
FG: Focus group
NSW: New South Wales
VCPS: Virtual clinical pharmacy service
WNSWLHD: Western NSW local health district
