# Supplementary material for: “This is streets ahead of what we used to do”: staff perceptions of virtual clinical pharmacy services in rural and remote Australian hospitals
Source: BMC Health Serv Res. 2021 Dec 4;21:1306. doi: 10.1186/s12913-021-07328-w (PMC8645070; doi:10.1186/s12913-021-07328-w)
Supplement: Supplementary file 1 — Additional file 1. [file 12913_2021_7328_MOESM1_ESM.docx]

VCPS Focus group questions

1. The virtual pharmacy has been operating for 3 months at this site. What was the implementation like? (Prompt – how were you informed about it? How it would work? How to refer patients?) (service delivery)
2. What is your experience of the virtual clinical pharmacy service? (Prompt - has the virtual clinical pharmacy service changed the way you work? What tasks are you required to do that are different? Describe what you like or don’t like about the service, how does it benefit clinicians, or does it cause any problems (Employee experience)
3. What was your experience of availability of a clinical pharmacist for medication review and medication advice prior to the virtual service? (Prompt- was the pharmacist approachable? (employee experience)
4. What benefits do patients and their cares experience as a result of the VCPS? (Prompt – Compliance with meds, understanding of meds, availability of pharmacist, ask for examples - Do you have any VCPS success stories you would like to share?) (Employee experience)
5. What about problems for patients? Can you give any examples? (Employee experience and service delivery)
6. How well do you think the structure and management of virtual pharmacy are working? Can you recommend any changes? (Prompt- What feedback have you received from the VCPS e.g. monthly data, in services, service rounding, one-on-one? How do you feel VCPS supports your work?) (service delivery)
7. What is your experience with the formal or informal education and advice provided by the Virtual Clinical Pharmacy Service? (service delivery/ experience – Are you happy with the level of support and education provided to develop skills in medication management)
8. Can you describe any challenges with the VCPS not already discussed?
9. Is there anything else you would like to add?
